# Process Induced Morphology Development of Isotactic Polypropylene on the Basis of Molecular Stretch and Mechanical Work Evolutions

**DOI:** 10.3390/ma12030505

**Published:** 2019-02-07

**Authors:** Sara Liparoti, Vito Speranza, Roberto Pantani, Giuseppe Titomanlio

**Affiliations:** Department of Industrial Engineering, University of Salerno—via Giovanni Paolo II, 132, 84084 Fisciano (SA), Italy; sliparoti@unisa.it (S.L.); rpantani@unisa.it (R.P.); gtitomanlio@unisa.it (G.T.)

**Keywords:** morphology, injection molding, numerical simulation, morphology prediction, shear layer

## Abstract

It is well known that under high shear rates polymers tend to solidify with formation of morphological elements oriented and aligned along the flow direction. On the other hand, stretched polymer chains may not have sufficient time to undergo the structuring steps, which give rise to fibrillar morphology. In the last decades, several authors have proposed a combined criterion based on both a critical shear rate and a critical mechanical work, which guaranties adequate time for molecular structuring. In this paper, the criterion, reformulated on the basis of critical values of both molecular stretch and mechanical work and adjusted to account for the unsteady character of the polymer processing operations, is applied to the analysis of a set of isotactic polypropylene injection molded samples obtained under very different thermal boundary conditions. The evolutions of molecular stretch and mechanical work are evaluated using process simulation. The results of the model reproduce the main characteristics of the morphology distribution detected on the cross sections of moldings, obtained under very different thermal boundary conditions, assuming that the critical work is a function of temperature.

## 1. Introduction

The crystallization morphology of polymers solidifying from flowing melts has been the object of several studies [1,2,3,4,5,6,7,8]. Many of them indicate that there is a critical shear rate, of the order of the inverse of Rouse time of the longest chains in the polymer, above which the morphology of the solidified polymer shows an ensemble of shish kebabs oriented along the flow direction, if the mechanical work accumulated afterwards is larger than a critical value [6,9,10]. The critical work guaranties the time required for kinetic processes finalized to create stable nuclei and to align the stable nuclei into rows, extending them along the flow direction until the microstructure is saturated. The higher the stress the faster those processes are [7].

It is well known that, during the injection molding process, oriented shish-kebab layers (also called shear layers) form within the final object cross section, and that the thickness of the shear layer changes remarkably depending on the processing conditions [11,12].

The results of the criterion for the formation of shish-kebab morphology were already favorably considered in comparison with the cross-section morphology distribution reported in the literature for isotactic polypropylene (iPP) injection molding samples, obtained under conventional injection molding conditions (CIM) [13]. In those cases, a critical molecular stretch parameter was recognized as more appropriate than the critical shear rate in order to obtain reliable predictions of the shear layer width. The evolutions of both the molecular stretch parameter and the mechanical work, accumulated after the critical stretch had been reached, were predicted adopting appropriate field equations for the description of temperature and flow fields. The effect of a stretch relaxation, while the mechanical work is accumulated, was disregarded in the previous work [13]. The constitutive equations for the rheological and crystallization behaviors (and their interactions) specifically identified for the adopted resin were also included in the model [14,15,16].

The aim of this paper is to identify a criterion based on the critical values of both a molecular stretch and the mechanical work and to apply it to the complete processing of the material to a final product. The aim of this paper is to identify a criterion based on the critical values of both a molecular stretch, and the mechanical work and to apply it to the complete processing of the material up to a final product accounting of unsteady conditions which in the injection molding can not be neglected since at the end of the filling step even a relaxation takes place. In order to verify the criterion against a set of injection molding conditions as wide as possible, the criterion was applied to moldings obtained with a procedure that allows a fast evolution of the cavity surface temperature during the process [17,18,19]. Such a procedure (by adopting appropriate cavity surface heating powers and times) allows the calibration of the morphology of the moldings all the way from a shear layer over most of the cross section to nearly completely spherulitic cross sections [19,20]. In order to evaluate the predictions of the model for crystallization into an oriented shish-kebab morphology, the evolutions of both the molecular stretch parameter and the mechanical work were evaluated using simulation of the injection molding process, accounting for cavity surface heating during the process. The overall objective was the optimization of the morphology inside the molded parts through modulation of the process parameters.

## 2. Materials and Methods

The simulation of the injection molding process was performed describing the evolution of the temperature field along both flow and thickness directions; to that purpose, convection, transverse conduction, and both crystallization heat and viscous heat generations [14,15,21] were accounted for. As mentioned in the introduction, the cavity surface was kept, during the process and until cooling, at temperatures intermediate between injection and mold temperatures. To this purpose, thin heating devices, made of several thin layers (one of which being the heating element operated by joule effect), were adopted. The heat transfer inside the heating devices was also simulated. 

Momentum balance was solved adopting lubrication approximation with a viscosity depending upon shear rate, $\dot{\gamma }$, through a Cross equation with zero-shear rate viscosity *η*_0_ (function of temperature, *T*, pressure, *P*, and crystallinity, *χ*) [14].

(1) $$\eta \left(T,P,\dot{\gamma },\chi \right)=\frac{\eta_{0}\left(T,P,\chi \right)}{1+{\left(\frac{\eta_{0}\left(T,P,\chi \right)\dot{\gamma }}{\tau_{R}}\right)}^{1-n}}$$

In Equation (1), *n* and *τ_R_* are material parameters. The viscoelastic nature of the polymer was considered using a relaxation time *λ* [14].

(2) $$\lambda \left(T,P,\chi ,\Delta \right)=\frac{\lambda_{0}\left(T,P,\chi \right)}{1+{\left(a\Delta \right)}^{b}}$$

The function of molecular stretch parameter *Δ*, whereas *a*, *b*, and *λ*_0_ are material parameters. The molecular stretch parameter was evaluated as the difference between the largest and the smallest eigenvalues of the molecular conformation tensor [22,23]

(3) $$\underset{=}{A}=3\frac{\left(\langle \underline{R}\underline{R}\rangle -{\langle \underline{R}\underline{R}\rangle }_{0}\right)}{\langle R_{0}^{2}\rangle },$$

where $\underline{R}$ is the end-to-end vector of a molecular sub chain. The evolution of the conformation tensor describing the sub chain population was described using Maxwell-type equation

(4) $$\frac{D}{Dt}\underset{=}{A}-{\left(\underline{\nabla }\underline{v}\right)}^{T}\times \underset{=}{A}-\underset{=}{A}\times \left(\underline{\nabla }\underline{v}\right)=-\frac{1}{\lambda }\underset{=}{A}+{\left(\underline{\nabla }\underline{v}\right)}^{T}+\left(\underline{\nabla }\underline{v}\right),$$

where $\underline{\nabla }$ $\underline{v}$ is the velocity gradient.

Mesomorphic and α crystallization processes, both competing for the same amorphous, were considered. The Nakamura equation [24] was adopted for the kinetics of the mesophase, the and Hoffman‒Lauritzen equation [25] was adopted for the crystallization toward the α phase.

(5) $$G\left(T\left(t\right),P,\Delta \right)=G_{0}\exp\left(-\frac{U}{R\left(T-T_{\infty }\right)}\right)\exp\left(-\frac{K_{g}\left(T+T_{m}\left(P,\Delta \right)\right)}{2T^{2}\left(T_{m}\left(P,\Delta \right)-T\right)}\right)$$

The effect of both pressure and flow on crystallization kinetics were accounted for by considering the crystallization temperature *T_m_* function of both pressure, and molecular stretch parameter, *Δ* [14,26]. 

The temperature and pressure evolutions obtained by the simulations were satisfactorily compared with experimental results. In addition, final crystallinity distributions inside moldings obtained with the iPP T30G were satisfactorily described [22]. In this work, the simulations were performed with the aim of analyzing the morphology distribution along the cross sections of moldings previously obtained and reported in [20]. The experimental information relevant to the simulation of those tests is summarized below.

Injection molding experiments were carried out adopting the iPP grade T30G (supplied by Lyondell Basell, Ferrara, Italy) previously characterized for rheology and crystallization kinetic [13,14,16]. The polymer was injected into a cavity having the thickness, width, and length of 1.50 mm, 12.7 mm, 110 mm, respectively. Five pressure transducers were placed along the flow path: one (P0) in the injection chamber, one (P1) in the runner close to the gate, and three (P2, P3 and P4) inside the cavity, 15 mm, 60 mm, and 105 mm downstream from the gate position, respectively. Thin heating devices were placed inside the mold, layered very close to the cavity surface (0.1 mm from it) in order to achieve a fast evolution of the cavity surface temperature during the injection cycle; the heating devices were made of several polymeric layers, one of them (thickness 0.05 mm) was electrically conductive being made of carbon black loaded poly(amide-imide). A fast thermocouple was allocated on the cavity surface in position P2. The injection molding tests were performed with 2.3 cm^3^/s average flow rate, 220 °C melt temperature, 25 °C overall mold temperature, 720 bar holding pressure, and 10–13 s holding time. 

The electrical power, P_e_, supplied to the heating devices determined the evolution of the cavity surface temperature toward a temperature level intermediate between mold and injection temperatures. In particular, with 4 W/cm^2^ or 9.5 W/cm^2^, the cavity surface reached about 80 °C or 150 °C, after about 6 s; in the following, these temperatures will be denoted as cavity surface temperature, T_cs_ (for each injection molding test). In order to avoid a cold contact of the polymer with the cavity surface, the electrical power in the heating devices was supplied in advance, in particular of a time t_a_ = 2 s, with respect to the time at which the flow front reaches the position P2 inside the cavity. The cooling steps were determined by de-activation of the heating devices.

Tests were performed adopting, for each heating power, the following heating times, t_h_, (after the cavity surface pre-heating): 0.7 s (namely the cavity filling time), 1.3 s, and 6 s. In addition, tests without activating the heating devices, namely Passive test, and tests where the heating devices were replaced by steel layers of the same geometry (namely Steel test) were carried out. All injection molding tests considered in this work are reported in Table 1.

Polarized optical micrographs (OM) of sample slices cut along the flow—thickness planes were obtained using a Olympus BX51 microscope (Olympus Italia S.r.l., Segrate, Italy) with crossed analyzer-polarizer. The flow direction of sample slices was rotated 45° with respect to the analyzer [20].

The slices were chemically etched following procedure proposed by Bassett [27], and analyzed with Atomic Force Microscopy (Bruker Dimension coupled with Nanoscope V controller) operating in tapping mode with a probe tip having 42 N/m spring constant, 300 kHz resonance frequency, and 7 nm radius (Bruker, Billerica, MA, USA) [20].

**Figure 1:**
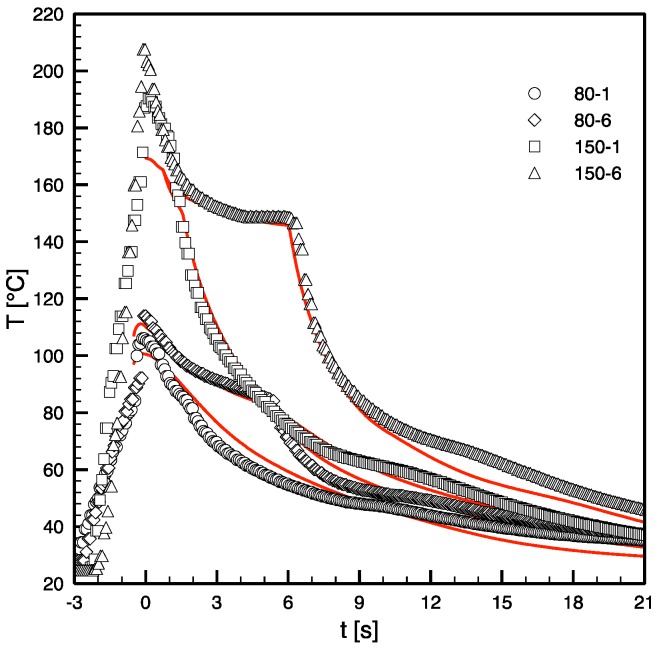
Comparison between experimental (symbols) and simulated (red full lines) cavity surface temperatures evolutions obtained for the tests 80-1, 80-6, 150-1 and 150-6.

**Figure 2:**
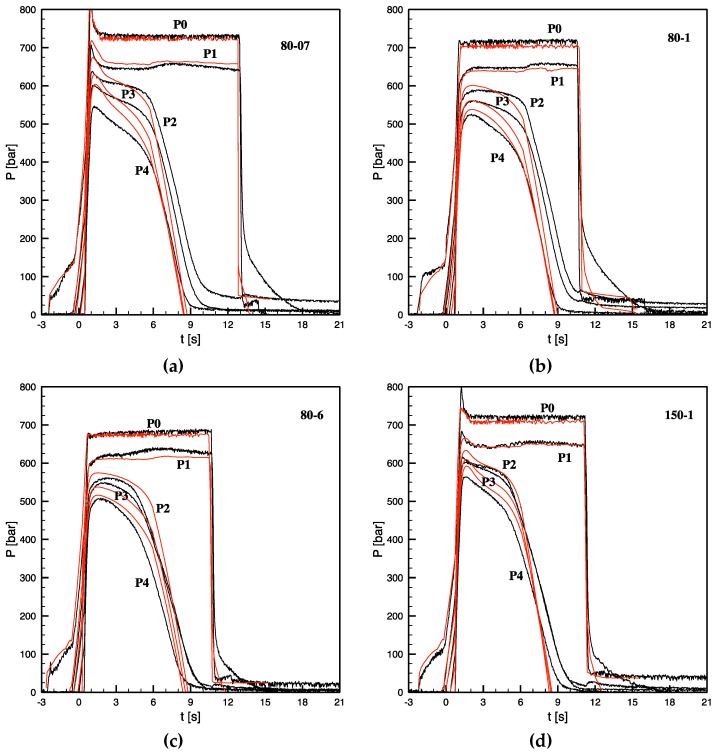
Comparison between experimental (black lines) and simulated (red lines) pressure evolutions for the tests carried out with heating powers 4 W/cm^2^ and 9.5 W/cm^2^ with different heating time as specified in single plot. (**a**) 80 °C and 0.7 s, (**b**) 80 °C and 1.3 s, (**c**) 80 °C and 6 s, (**d**) 150 °C and 1.3 s.

Figure 1 and Figure 2 show comparisons between some simulation and experimental results for evolution of surface temperature in position P2 and for pressure distributions during some of the tests listed in Table 1.

As mentioned above, the heating of the mold surface started in advance with respect to the first contact of the polymer in position P2, and, in Figure 1, the value of time is set to zero at the polymer contact in position P2. The electrical power was selected on the basis of the desired final temperature. When the polymer reaches position P2, it undergoes a jump up by effect of the temperature of the hot polymer, which was injected at 220 °C. After about 6 s, a nearly steady temperature on the cavity surface was recorded. Sample cooling takes place by effect of the temperature difference between the sample and the cavity surface; cavity surface temperature decreases to the value of the mold at the heating device de-activation.

The main characteristics of the experimental temperature evolutions on the cavity surface were reproduced by the simulation. The differences are mainly related to the description of the sample cooling and, in particular to the inflection zones along the experimental curves, at temperatures below 80 °C. These inflections have to be related to concentrated development of the latent heat of crystallization, occurring at the detachments of the sample from the cavity surface, which were not accounted for in the simulation.

Figure 2 shows the comparison between experimental and simulated pressure evolutions along the flow path, for some of the tests considered in this paper. Similarly to the temperature evolutions reported in Figure 1 and in the plots reported in Figure 2, the time t = 0 s corresponds to the first contact of the melt in position P2. At the end of the filling step, the injection chamber pressure, P0, was found to range between 450 bars and 340 bars upon a change of the cavity heating power from 4 W/cm^2^ or 9.5 W/cm^2^. At the end of the filling step, the pressure undergoes a jump toward the holding pressure (which was kept at 720 bars for all tests).

After filling, the density increase, determined by both cooling and crystallization processes taking place inside the cavity, is compensated by the packing flow. The packing flow essentially keeps the pressure constant inside the cavity, as long as it is not hindered by the gate sealing process. When the gate sealing process starts to have an impact on the packing flow, the pressure drops through the gate (essentially between P1‒P2). When the gate sealing process starts, to have an impact on the packing flow, the pressure drops through the gate (essentially between P1–P2) undergoes a fast increase of the enhancement rate process, which for all tests considered in this work takes place between 5 s and 6.5 s. 

The pressure in position P1 drops to zero when the holding pressure is released in the injection chamber, unless, somewhere between positions P0 and P1, the process of cross-section solidification is already sufficiently advanced to significantly slow down the back flow from P1 to P0. Indeed, for all the plots reported in Figure 2 the pressure in P1 drops instantaneously to a value small but still considerable (about 100 bars), soon after it slowly reduces to zero. 

The main experimental characteristics of pressure evolutions, in particular, the pressure growth during the filling step, both pressure levels at beginning of the packing step, and gate sealing times, are also essentially nicely reproduced by the simulations. The slopes of the pressure curves during cooling are only slightly overestimated. The existence of an advanced (not complete) process of cross-section solidification between the injection chamber and position P1, at the end of the holding time, is also reproduced, as demonstrated by the fact that the pressure does not completely drop to zero at the holding pressure release. 

In conclusion, the comparisons between experimental acquisitions and simulation results show that the main characteristics of the thermo-mechanical histories, and thus of the phenomena involved during the process, are reproduced by the simulation. This is a strong indication that relevant phenomena taking place during the process were satisfactorily described, thus accrediting the use of the simulation results for the application of the criterion for the formation of fibrillar morphology.

## 3. The Criterion for Crystallization into Fibrillar Morphology

The idea that the criterion for the attainment of fibrillar morphology, as found in the shear layer of injection molded objects, has to be based on the achievement of an adequate (critical) amount of mechanical work spent with shear rates above a critical value was proposed several years ago [9,10,13,28,29,30]. The critical value of shear rate was identified to be of the order of the inverse of the Rouse number, because it has to be able to sufficiently stretch the macromolecules. The critical mechanical work stands there to allow for kinetic processes related to the stretching of long molecules, creating stable shish nuclei aligned into rows, upon which the bulk of the material can crystallize as kebabs, transforming it into a fibrillar structure; the higher the stress the faster are those processes.

Reformulating the criterion in terms of the molecular stretching parameter *Δ* [13] is equivalent, or even a step ahead, with respect to the initial formulation in terms of shear rate. In other words, for each polymer, there is a critical amount of mechanical work, W_c_, which, once accumulated in the presence of a molecular stretch, in other words, for each polymer, there is a critical amount of mechanical work, W_c_, which, once accumulated in presence of a molecular stretch, *Δ* equal or larger than the critical stretch, Δ_c_, determines the crystallization into the shish—kebab morphology. The authors believe that the larger the value of the stretch (above the critical value), the larger the density of molecules will be that will be able to act as nucleation seeds for shish formation. If the molecules have sufficient available time (assured by the accounting of mechanical work), they will arrange themselves into a mechanically efficient morphology, and the elements thickness would be determined by the density of the active nucleation seed, after lateral growth.

If while the mechanical work increases, there is some relaxation, which brings the stretch parameter below its critical value, Δ_c_, the formation of the fibers remains, at least partially, compromised: even if the formation of a structure propaedeutic to the ordered and aligned shish-kebab morphology had begun, a morphology more or less different (depending on the extent of the stretch deviation), would form. If the stretch recovers its critical value, the accounting of the mechanical work will have to start again and, only if it reaches its critical value, W_c_, a well-organized, aligned, and ordered structure will form. One would expect that the amount of deviation from a wel-organized and aligned structure would depend upon the amount of deviation from the criterion.

## 4. Stretch and Mechanical Work Distributions

**Figure 3:**
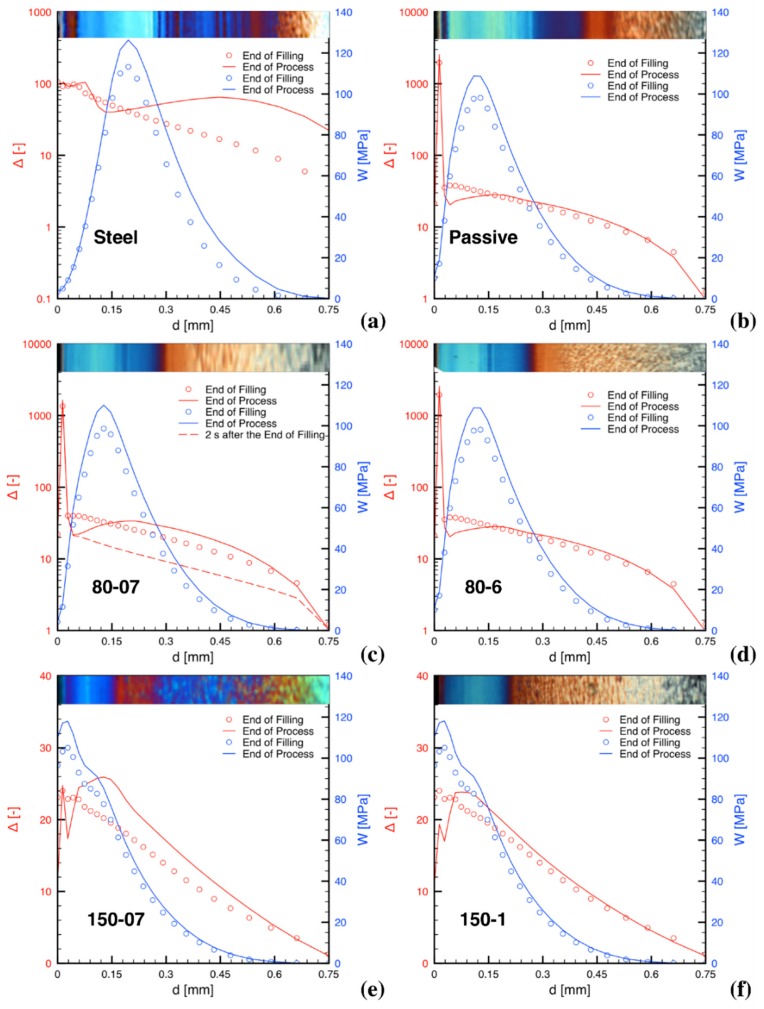
Molecular stretch distribution (namely Δ in red) and integral of mechanical work (namely W in blue) as calculated by the simulation for tests with different T_cs_ and heating times: Steel (**a**); Passive (**b**); 80 °C and 0.7 s (**c**); 80 °C and 6 s (**d**); 150 °C and 0.7 s (**e**); 150 °C and 1.3 s (**f**). Cross section distributions in position P2 at the end of filling (symbols) and at the end of the process (full lines). The Δ distribution 2 s after the end of filling (red dashed line) is also reported for the test 80-07 (**c**).

The distributions of both the molecular stretch, *Δ*, and the integral of the mechanical work, *W*, both at the end of the filling step, and at the end of the process, are reported in Figure 3 for some of the injection conditions considered in this paper; in particular, the following conditions are considered: Steel, Passive, 80-07, 80-6, 150-07 and 150-1. In addition, the corresponding optical micrographs of the final molding cross-section morphologies in position P2 are reported on the top of each plot. The optical micrographs give some information about the cross-section morphology distribution, but several aspects need deeper analysis to be identified. Certainly, the surface layer, appearing as a dark layer close the sample surface, when present, can be identified in the optical micrographs. In the literature, it was pointed out that this layer, unlike shear layer, is characterized by the presence of unaligned elements [31]. Furthermore, an area, often rather wide, with spherulitic character can be identified on the symmetry plane of the sample. More difficult to detect using optical microscopy is the shish-kebab morphology, especially if (and when) the oriented elements are very thin. Under some processing conditions, between the shish kebab and the spherulitic morphology, there is a transition region, where elongated elements, having thickness ranging from about 4 μm up to 6 μm, are aligned (although sometimes not perfectly) along the flow direction. Depending upon operating conditions, the thickness of the transition region can be very wide or it can even disappear; sometimes the morphology of this region can (especially where the elements are thick) be detected using optical microscopy. What is difficult to identify using optical microscopy are both the borders of this region, which, therefore, remain without a quantitative definition. 

A comparison of *Δ* and *W* distributions for the Steel and Passive tests shows that main differences concerning the plot of *Δ* can be ascribed to the higher cooling rate of the Steel test with respect to the Passive test. Close to the sample surface, the material, due to the very high cooling rate that occurs during its first contact with the mold, cannot be deformed before solidifying, thus the peak showed in the Passive test vanishes. Moreover, the higher cooling rate in the Steel test enhances the effect on the stretch of the packing flow, thus the final stretch curve shows a more pronounced maximum and higher stretch values in the region close to the sample midplane than in the Passive test.

The boundary conditions difference between the Passive and the 80-07 tests is small, being limited to the cavity surface temperature only during the filling step; consequently, the corresponding two plots of *W* and *Δ*, reported in Figure 3, are very similar. By comparing the distribution curves of the same variable in the two tests, small differences can be identified; they are consistent with a slightly faster cooling for the Passive sample. The plot corresponding to the condition 80-6 is similar to 80-07, except for the final Δ distribution, which undergoes an additional relaxation with respect to the 80-07 test.

In order to point out that soon after the end of the filling step there is a stretch/stress relaxation, a fifth curve (the dashed red one) reporting the *Δ* distribution, calculated about two seconds after the end of the filling step, has been included in the plot related to the condition 80-07. Considerable relaxation, with respect to the *Δ* distribution, at the end of the filling step is clearly detectable. During such a relaxation, an additional stretch build up starts to set in as a consequence of the packing flow. The final stretch distributions also include eventual further relaxation determined by long cavity surface heating, if present. The packing steps and the eventual final relaxation determine the shape of the final stretch curve.

The tests 150-07 and 150-1, obtained with the highest heating electrical power, show values of the final stretch distribution much smaller than those evaluated in the other conditions. This is due to a packing flow poorly effective in stretching molecules, whereas the relaxation is faster. The stretch peaks at the surface are still present, although their values are orders of magnitude smaller than in the other tests due to the high temperature (150 °C) during both the filling and the packing steps. 

The comparison of the stretch distributions obtained for the tests that adopt different heating powers confirms that the higher the heating power is the faster the stretch relaxation rate is. Indeed, the stretch relaxation rate with the power of 4 W/cm^2^ (which allows reaching 80 °C) certainly appears slower than with the heating power of 9.5 W/cm^2^ (which allows reaching 150 °C); in this case in fact, a large decrease of *Δ* occurs in a very short time (only about 0.6 s).

As far as the integral of the mechanical work is concerned, close to the cavity surface the contribution of the filling step appears dominant; the contribution of the packing step (although still minor) is larger in the region of the maximum; beyond the maximum the packing step gives a contribution which continuously decreases toward the sample midplane, and the decrease is faster when either the cavity surface temperature or the heating time increase.

It is worth pointing out that the plots reported in Figure 3 are not sufficient to clarify if, according to the criterion illustrated above, at the end of the process, in a given position on the cross section, the morphology will be fibrillar or not. Indeed, the criterion for fibrillar morphology specified above clarifies that the fraction of mechanical work accumulated before the stretch reaches its critical value Δ_c_ has to be disregarded in the account and, furthermore, if by effect of a relaxation the stretch decreases below its critical value, the accounted mechanical work disappears (has to be cancelled). 

## 5. Final Cross Section Morphology as Determined by Molecular Stretch and Mechanical Work Evolutions

By essentially applying the criterion aforementioned with Δ_c_ and W_c_ equal to 7 and 10 MPa respectively, the morphologies of some moldings obtained using CIM with the same iPP adopted in this paper was correctly predicted [13]; therefore, these critical values will be the starting point of the following analysis.

**Figure 4:**
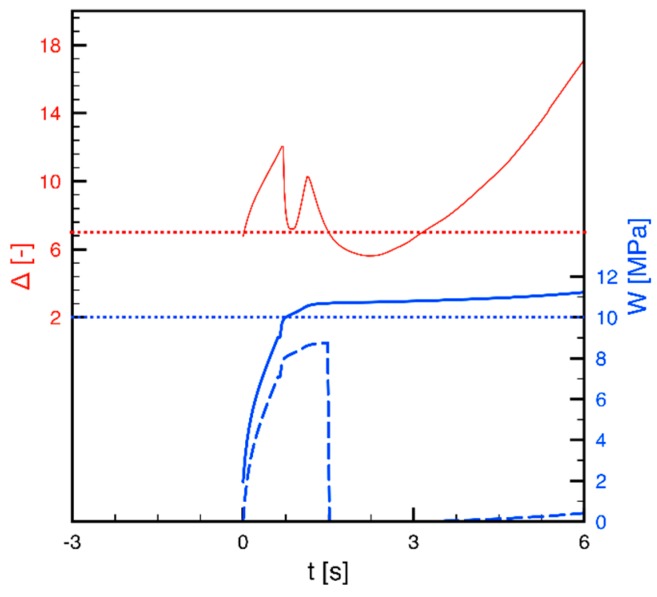
Predicted evolution of the molecular stretch (solid red line) and mechanical work (solid blue line) at 0.44 mm from the cavity surface for the test 80-07. The dashed blue line represents the effective mechanical work, W_e_.

Let us now combine the molecular stretch and the mechanical work evolutions for a case far from the surface, in particular, in position P2 and at 0.44 mm from the cavity surface for the test 80-07. For this case, the molecular stretch and the mechanical work evolutions are reported in Figure 4. The red line is the molecular stretch, whereas the full blue line is the total work accumulated from the beginning of the process. The horizontal red and blue dotted lines represent the critical value of the molecular stretch and the mechanical work respectively, namely 7 and 10 MPa. However, according to the criterion adopted in this paper, the mechanical work accumulated before the molecular stretch has reached its critical value has to be disregarded. We will denote as “effective mechanical work”, W_e_, the work done when molecular stretch is beyond its critical value. W_e_ is also plotted in Figure 4 as a dashed blue line. Since the molecular stretch at about 1.4‒1.5 s, as a consequence of the relaxation taking place after filling, drops below its critical value of 7, according to the criterion adopted in this paper, starting from that time, the effective work already accumulated has to be disregarded. Thus, its accounting drops to zero, and, from that time, it starts to increase again at about 3 s when the molecular stretch again overcomes its critical value. However, the work accumulated during the packing step is not sufficient to reach an effective critical work, in this case 10 MPa. Consequently, the condition for fibrillar morphology will not be achieved, although the stretch, and the integral of the overall mechanical work at the end of the process both reach their critical values.

**Figure 5:**
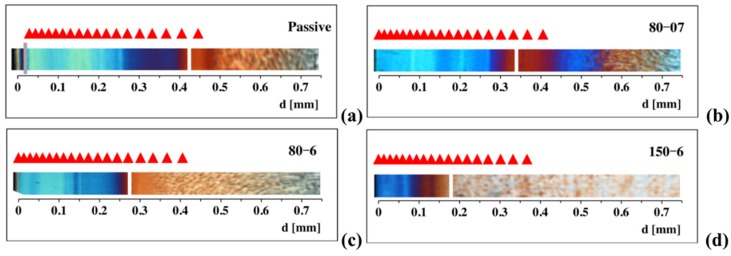
Comparison between experimental observation for the layers with oriented morphological elements of thickness not larger than 4 μm [20] (white marks) and predictions of the criterion obtained with Δ_c_ and W_c_ equal to 7 and 10 MPa respectively (red triangles).(**a**) Passive test; (**b**) 80-07 test; (**c**) 80-6 test; (**d**) 150-6 test.

For some of the tests considered in this paper, the results, obtained applying the criterion clarified in Figure 4 (with Δ_c_ and W_c_ equal to 7 and 10 MPa respectively), for the thickness of the layer characterized by ordered and aligned elements are reported in Figure 5 as red triangles above corresponding optical micrographs. 

The main effects of the operating conditions on the different characteristics of the cross section experimental morphology distribution of the same moldings considered in this paper have recently been investigated in detail using Atomic Force Microscopy (AFM) [20]. On the basis of that AFM analysis, a layer including both shish kebab and oriented and aligned morphological elements of thickness not larger than 4 μm was identified; its boundary is reported as a white mark in each of the optical micrographs of Figure 5. Moreover, on the optical micrograph of the Passive test an additional grey mark is reported to identify the surface layer.

**Figure 6:**
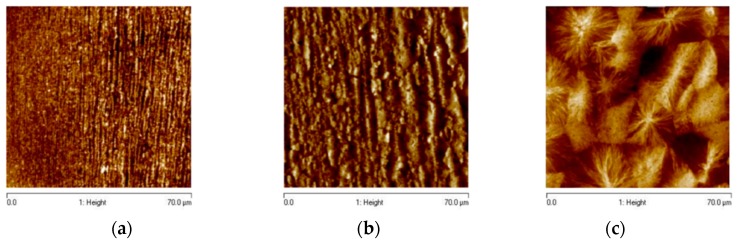
Atomic Force Microscopy (AFM) images over an area of 70 μm × 70 μm at 0.25 mm (**a**), at 0.38 mm (**b**), and 0.7 mm (**c**) from the sample surface for the test 80-07. At 0.25 mm, morphological elements aligned along the flow direction tightly packed can be observed. At 0.38 mm, the average diameter of the morphological elements aligned along the flow direction range from about 4 μm to about 6 μm. At 0.585 mm, spherulitic structures cover the whole investigated area.

Examples of different morphologies developed along the sample cross section are reported in Figure 6a–c. The AFM height images reported in Figure 6a–c were acquired for the 80-07 test at a distance of 0.25 mm, 0.38 mm (indicated by the white mark in the optical micrograph of Figure 5b), and 0.585 mm from the sample surface, respectively. Figure 6a shows that the blue layer in Figure 5b is a highly oriented layer characterized by tightly packed thin structures aligned along the flow direction; their thickness increases with the distance from the surface. Figure 6b shows that in correspondence of the white mark of Figure 5b there is a thickness transition (from about 4 μm to about 6 μm) of the oriented and aligned morphological structures. Figure 6c shows that on increasing the distance from the sample surface only un–oriented spherulitic structures can be detected.

The comparison between predictions of the criterion and experimental results for the positions and width of the layers presenting shish-kebab and elements ordered and oriented along the flow direction can be considered positive for the Passive test and the 80-07 test (Figure 5a,b respectively). Vice versa, when the heating time and the heating power increase the comparison cannot be considered satisfactory, as shown in Figure 5d for the test 150-6, for which the results of the criterion gave rise to a width more than double with respect to the experimental results. 

**Figure 7:**
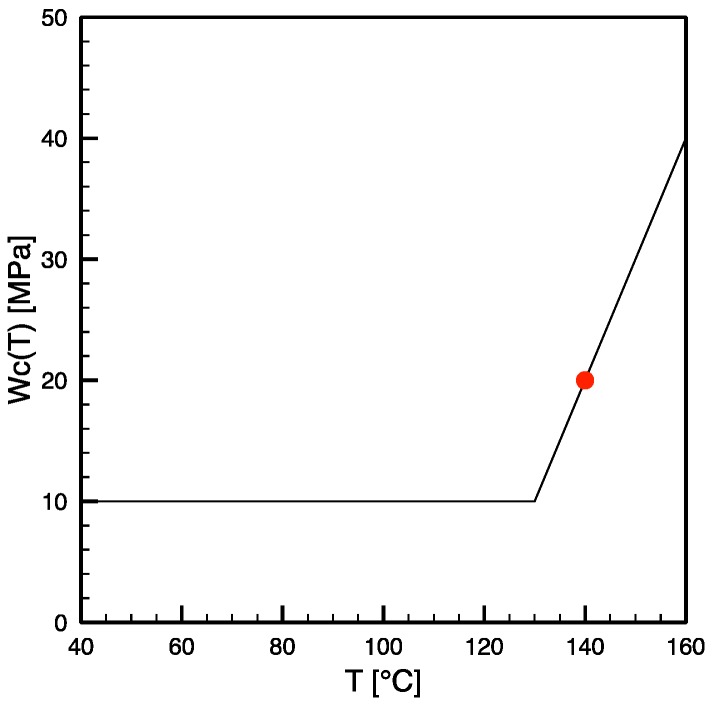
Critical work as function of temperature adopted for the results of simulations reported in Figure 8; the red dot represents the experimental results at 140 °C obtained using the iPP T30G adopted for the injection molding tests considered in this paper.

On the other hand, experimental results reported by the Group of Sheffield University [10] indicated an increase of the critical work with the temperature for a low density polyethylene and a polypropylene-ethylene random copolymer. Furthermore, the results of an experimental analysis of the effect of shearing on the shish-kebab crystallization carried out at 140 °C using a Linkam cell on the same iPP adopted in this paper gave rise to a critical work of 20 MPa, rather than the 10 MPa adopted for the comparison reported in Figure 5. In addition, it has been shown [13] that the value of the critical work, W_c_, determines not only the thickness of the surface layer (when present), but often it is also the controlling parameter for the thickness of the oriented layer (for instance, for the case 80-07 considered in Figure 4). One can thus expect that the increase of the mechanical work is consistent with the objective of obtaining predictions of thinner oriented layers at larger temperatures, as shown by the experimental results. In conclusion, the predictions for thickness and location of oriented layers of the injection molded samples considered in this paper were calculated again adopting critical stretch Δ_c_ equal to 7 (as for the comparisons reported in Figure 5), and a critical work W_c_ function of the temperature as shown in Figure 7, namely, equal to 10 MPa at low temperature, and linearly increasing for temperatures larger than 130 °C, considering that W_c_ was experimentally found to be 20 MPa at 140 °C (reported as a red dot in Figure 7).

**Figure 8:**
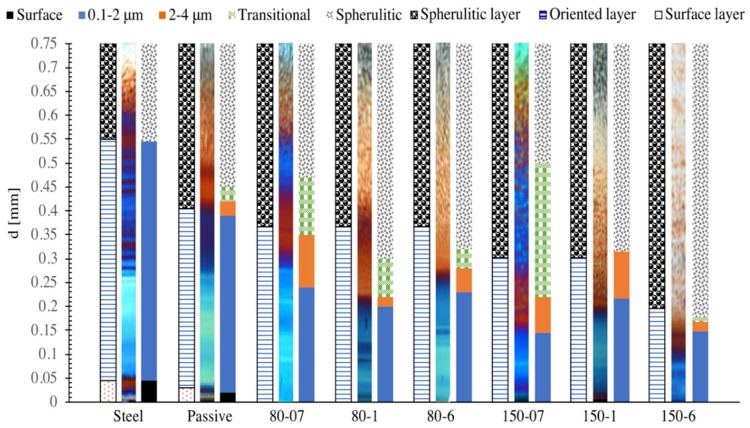
Comparison between experimental morphology distribution along the cross sections of the injection molded samples under the conditions listed in Table 1. Three bars are reported for each of the injection conditions, all of them refer to the morphology distribution in position P2: the central bar is the optical cross section micrograph; the bar on the left reports the predictions of the criterion adopted in this paper for the thicknesses of the surface layer, when present, of the oriented morphological layer and of the spherulitic layer; the bar on the right reports the width and characteristics of the morphological layers as detected using AFM analysis reported in [20]. Each color of the bar on the right corresponds to the thickness of the morphological elements as specified in the legend.

For each of the eight injection molded tests considered in this work, the predictions of the criterion (based on Δ_c_ = 7 and W_c_ as shown in Figure 7) for the morphology along the cross section in position P2 are compared in Figure 8 with a synthesis of the corresponding experimental morphology distribution as detected by the AFM analysis reported in [20]. The corresponding polarized optical micrographs are reported in the figure. In particular, in the synthesis of the experimental morphology, layers of different colors, as indicated in the legend, correspond (after the surface layer which is black) to oriented morphological elements having thicknesses growing with the distance from the sample surface in the intervals 0.1–2 μm (blue) or 2–4 μm (orange) or, sometimes, a transitional layer toward a spherulitic morphology with elements of thickness ranging in the interval 4–6 μm (green); finally, a spherulitic layer was always present up to the sample midplane. Only the layer 0.1–2 μm and the spherulitic layer were found in all injection molding conditions, details of which are given below. Within the interval 2–4 μm, and especially within the interval 4–6 μm, when present, the morphology characteristics were found to gradually change their order and homogeneity. In particular, the order and the thickness of the elements were found to decrease, and the element thicknesses were found to increase with the distance from the sample surface including more and more cylindritic and sometimes small spherulitic structures.

In most of the cases, the evolution from oriented elements to the spherulitic morphology was found to take place within a transitional layer of width of the order of only 70 μm. For the Steel test the evolution took place even and directly from the layer which includes aligned structures with thicknesses ranging between 0.1 μm–2 μm to the spherulitic layer. Only the case of 150-07 showed a much more extended (nearly one half of the sample thickness) transitional layer; this has to be related to the intensity of the packing flow, which, in turn, is determined by the amount and rate of cooling and crystallization after filling. Indeed, in the case 150-07, the packing flow is expected to have had the maximum intensity since during filling only a moderate cooling takes place, and crystallization has not started yet (except for small percentages at the surface). To a smaller extent, similar observations can be made in relation to the 80-07 sample, which is second as far as width of the transitional layer.

As far as the comparison, shown in Figure 8, between experimental results and the results of the criterion for oriented morphology distribution on the molding cross section, it appears that, with the choices described above for the critical stretch and for the critical work, the surface layer widths for the Steel and Passive tests are correctly predicted, and the absence of the surface layer in all the other tests is also properly described. Furthermore, simulations reproduce, within a reasonable approximation, the additional widths from the surface layer (when present) up to the positions within which oriented and aligned morphological elements of average diameter up to 4 μm were identified [20]. The discrepancy is smaller than 15% (i.e., smaller than 40 μm) for all cases, except for the cases 80-1, 80-6, and 150-07, for which the discrepancies are 30% for the first one and 20% for the other two conditions. It has to be considered that the approximations of the comparison reported in Figure 8 are the result not only of the approximations of the application of criterion for the formation of ordered morphological structures aligned along the flow direction, but they also include all approximations within the model adopted for the calculation of the molecular stretch and the mechanical work evolutions. For instance, it would be sufficient that one of the parameters (as, e.g., the ones determining the dependence of the viscosity or of the relaxation time upon pressure or temperature) is slightly either over or under estimated to determine a significant effect on the evolutions of both the molecular stretch and the mechanical work distributions and consequently on the final cross-section morphology. Moreover, it must be pointed out that the predictions of both the molecular stretch and the mechanical work evolutions and consequently the width of the oriented shear layer observed in the final cross section of the samples are sensitive to the distance from the gate of the cavity. A difference of some millimeters (a few percent of the cavity length) in the position along the flow direction could significantly reduce the discrepancy between predictions and experimental observations. Finally, it has to be pointed out that, as mentioned above, the criterion for the crystallization into fibrillar morphology, in the format proposed in this paper, is still schematic; namely, it is an on/off model. Indeed, it does not consider intermediate morphologies when only a fraction (possibly high) of the critical mechanical work is achieved or the critical work is achieved while the stretch was not continuously above its critical value. The transition zones could be related to situations of this kind.

A further step toward a deep understanding of the morphology distribution along the sample cross section could be achieved by considering the stretch distribution along the oriented layer. In the literature [7,32,33,34] it was affirmed that the density of the shish increases with the increase of the shear (and thus of the stretch) experienced by the polymer. Thus, the critical stretch would be related (when and where the critical mechanical work is reached) to the minimum of shish density within the oriented layer. Such a minimum determines the minimum density of the oriented structures and therefore the maximum thickness (by effect of the lateral growth allowable) that the oriented structures can achieve. A change of the critical stretch would also induce a modification of the prediction for the width of the layer characterized by oriented structures. Furthermore, being the criterion for the fiber formation respected inside the oriented layer, the excess of stretch with respect to the critical value can be considered. This excess assumes different values along the layer, and it is non–negative. The local values of the excess of stretch should be coherent with the thickness distribution of the oriented structures and they could identify the local thickness of the oriented structures once the correlation between stretch and density of oriented and aligned structures is identified. This should be taken as the next goal.

## 6. Conclusions

In this paper, a criterion for the polymer crystallization into highly oriented morphological elements aligned along the flow direction was applied to the analysis of a set of isotactic polypropylene injection molded samples obtained under very different thermal boundary conditions. A formulation of the criterion in terms of critical values of both a molecular stretch parameter and the mechanical work was adopted, starting from a critical amount of mechanical work carried out when the stretch is above its critical value. In order to also take into account the fact that polymer processing operations are performed under unsteady conditions (and the injection molding process is typically unsteady), the previous formulation of the criterion was modified with the following specification: once the critical stretch is reached, if it relaxes below its critical value, the accounting of the mechanical work has to be canceled and started again.

In order to apply the criterion to the iPP injection molding samples, the evolutions of both the stretch and the mechanical work distributions were evaluated using process simulation. Taking the critical mechanical work function of temperature, as indicated by results of literature isothermal experimentations, gave rise to a satisfactory description of the morphology developed along the molding cross sections for the tests considered in this paper. In particular, widths and positions of surface (when present) and oriented layers were correctly described confirming that predictions exhibited essentially the same dependence upon the process conditions (heating power and heating time) shown by the experimental observations.

The criterion, in its actual on/off formulation, does not include the existence of intermediate transitional layers between the highly oriented and the spherulitic layers. In most of the cases, these transitions took place within very thin zones; however, under special processing conditions, the transitional zone can be wide. The possibility of transitional layers could be included in the criterion by releasing its on/off approach; namely, by considering the cases when either the mechanical work or the molecular stretch do not completely satisfy their critical conditions.

## Figures and Tables

**Table 1:** Injection molding operating conditions.

Test Run	P_e_ (W/cm^2^)	T_cs_ (°C)	t_h_ (s)	t_a_ (s)
Steel	0	25	0	0
Passive	0	25	0	0
80-07	4	80	0.7	2
80-1	4	80	1.3	2
80-6	4	80	6	2
150-07	9.5	150	0.7	2
150-1	9.5	150	1.3	2
150-6	9.5	150	6	2
